# The GLP-1 journey: from discovery science to therapeutic impact

**DOI:** 10.1172/JCI175634

**Published:** 2024-01-16

**Authors:** Daniel J. Drucker

**Affiliations:** Department of Medicine, Lunenfeld-Tanenbaum Research Institute, Mt. Sinai Hospital, University of Toronto, Toronto, Ontario, Canada.

Over several decades the *JCI* has published key advances in our understanding of glucagon-like peptide 1 (GLP-1) biology. The first incretin peptide characterized in the 1970s, glucose-dependent insulinotropic polypeptide (GIP), was isolated from porcine gastric extracts. Subsequently, the sequence of GLP-1 was identified following the cloning of the glucagon cDNAs and genes, soon followed by the demonstration that GLP-1 potentiated glucose-dependent insulin secretion in cells, animals, and humans (comprehensively reviewed in Drucker, et al.; ref. 1).

## Incretin action in islets and implications for diabetes

The findings that the acute insulinotropic actions of GLP-1, but not GIP, were relatively preserved in people with type 2 diabetes (T2D) (2) focused greater attention on the therapeutic potential of GLP-1, ultimately supporting multiple clinical development programs for GLP-1 receptor (GLP-1R) agonists (GLP-1RA). Physiologically, the essential roles of incretin receptors for glucose homeostasis have been demonstrated in single and double incretin receptor knockout mice. *Glp1r*^–/–^ mice, and, to a greater extent, *Glp1r*^–/–^:*Gipr*^–/–^ mice, exhibit defective glucose-stimulated insulin secretion, subnormal upregulation of insulin gene expression in response to high-fat diet (HFD) feeding, and impaired glucose tolerance (3, 4). In contrast, *Gipr*^–/–^ mice exhibit greater resistance to diet-induced obesity, relative to *Glp1r^–/–^* mice (4). The physiological importance of GLP-1R signaling has also been revealed in humans treated with GLP-1 receptor agonists such as exendin(9-39). Schirra and colleagues infused exendin(9-39) into healthy male human subjects, under euglycemic or hyperglycemic conditions, with or without concomitant i.v. administration of GLP-1 or GIP (5). Exendin(9-39) blocked the stimulation of insulin and the inhibition of glucagon secretion in the presence of exogenous GLP-1 administration but had no effect on the insulinotropic actions of GIP. Importantly, infusion of exendin(9-39) alone increased levels of plasma glucagon under conditions of both euglycemia and hyperglycemia, and decreased levels of plasma insulin when the glucose was elevated. Collectively, these findings revealed the essential physiological actions of GLP-1R and GIPR signaling for islet hormone secretion in mice and humans (5).

Among the holy grails of human islet research is the identification of methods to safely and effectively stimulate replication of human islet β cells. Dai and colleagues studied the uncoupling of GLP-1 responses linked to cell proliferation from those that potentiate glucose-dependent insulin secretion in juvenile versus adult human islets (6). Exendin-4 stimulated glucose-dependent insulin secretion in both juvenile and older adult human islets. However, examination of the proliferative response identified age-associated impairments in components of the calcineurin/NFAT signaling pathway that were responsive to exendin-4 in juvenile, but not in adult, human islets (6).

As GIP and GLP-1 exert their actions through structurally similar G protein coupled receptors, the differential mechanisms underlying preserved GLP-1, but not GIP, insulin stimulatory responses in diabetic β cells have remained enigmatic. Oduori and colleagues probed this anomaly in studies of mice and both murine and human islets exposed to hyperglycemia, and determined that a Gs/Gq signaling switch in β cells arises following exposure to sustained hyperglycemia (7). Notably, GLP-1 but not GIP, is able to activate both Gq and Gs, while GIP seems only to activate Gs, suggesting a possible mechanism for the diminished insulinotropic response to GIP in diabetic β cells.

## GLP-1 and the reduction of food intake

Following the demonstration that intracerebroventricular administration of GLP-1 inhibited food intake in mice and rats, treatment of animals with peripherally administered GLP-1RA was associated with reduction of food intake and weight loss (1). Flint and colleagues examined the effects of acute GLP-1 infusion on sensations of hunger and satiety in healthy human volunteers. GLP-1 infusion increased sensations of fullness and satiety and reduced solid food intake after breakfast and lunch (8). Observations in those treated with GLP-1RA subsequently confirmed weight loss in people with T2D, and later obesity (1).

Understanding the mechanisms underlying the anorexic effects of GLP-1 is of great interest. The GLP-1R is widely expressed in multiple regions of the rodent and human brain, and activation of GLP-1R^+^ neurons in the hypothalamus and brainstem reduces food intake and promotes weight loss. Chemogenetic activation of murine preproglucagon neurons in the hindbrain reduces food intake and metabolic rate and suppresses hepatic glucose production in normal mice (9). Activation of these GCG neurons in HFD-fed mice revealed a persistent reduction of food intake and body weight, without changes in glucose homeostasis or stress responses. Hence, this population of GCG neurons is likely important for fine tuning the control of food intake, but less essential for the control of whole-body glucose homeostasis. Furthermore, the relative importance of endogenous GLP-2 versus GLP-1 or glucagon as orchestrators of these chemogenetic responses was not determined and is clearly less important for weight control relative to pharmacological actions of the same peptides.

Sisley and colleagues used mouse genetics to inactivate the *Glp1r* in the mouse brain, demonstrating that the acute anorectic and chronic weight loss–inducing pharmacological actions of GLP-1RA required GLP-1R expression in the central nervous system (10). In contrast, loss of GLP-1Rs in the central or autonomic nervous system did not impact the physiological control of food intake or body weight, even under HFD conditions (10). These findings emphasize the robust GLP-1R–dependent pharmacological induction of weight loss, yet a comparatively modest importance of basal GLP-1R signaling for food intake or long-term energy homeostasis (1).

Secher and colleagues studied the importance of hypothalamic GLP-1R signaling for the anorectic actions of liraglutide in mice. Injection of fluorescent liraglutide labelled neurons in circumventricular organs, as well as the arcuate nucleus, showed brain uptake of labelled liraglutide was abolished in Glp1r^–/–^ mice, showing that brain uptake of liraglutide is dependent on the canonical GLP-1R (11). GLP-1 directly stimulated populations of POMC/CART neurons and inhibited the activity of neuropeptide Y+ and agouti-related peptide (AgRP) neurons. It is now appreciated that multiple regions within the hypothalamus, brainstem, and beyond, transduce pharmacological GLP-1R-dependent signals in the brain to reduce food intake, enabling weight loss with chronic administration of GLP-1RA (1).

Rupp et al. used single nucleus RNA-Seq to identify a population of GABAergic *Glp1r*-expressing LepRb neurons exhibiting robust expression of leptin-regulated genes in the mouse hypothalamus (12). Mice subjected to fasting followed by refeeding exhibited increased FOS immunoreactivity in dorsomedial hypothalamic *Glp1r* neurons with a distribution overlapping with that exhibited by LepRb^+^Glp1r^+^ neurons. Activation or deletion of *Lepr* in these neurons revealed an essential role for this neuronal population in the basal control of food intake. Similarly, selective rescue of the GLP-1R in this hypothalamic neuronal population of *Glp1r*^–/–^ mice restored an anorexigenic response to GLP-1R agonism, evident following acute liraglutide administration (12).

## GLP-1 actions beyond insulin secretion and body weight

**Figure 1:**
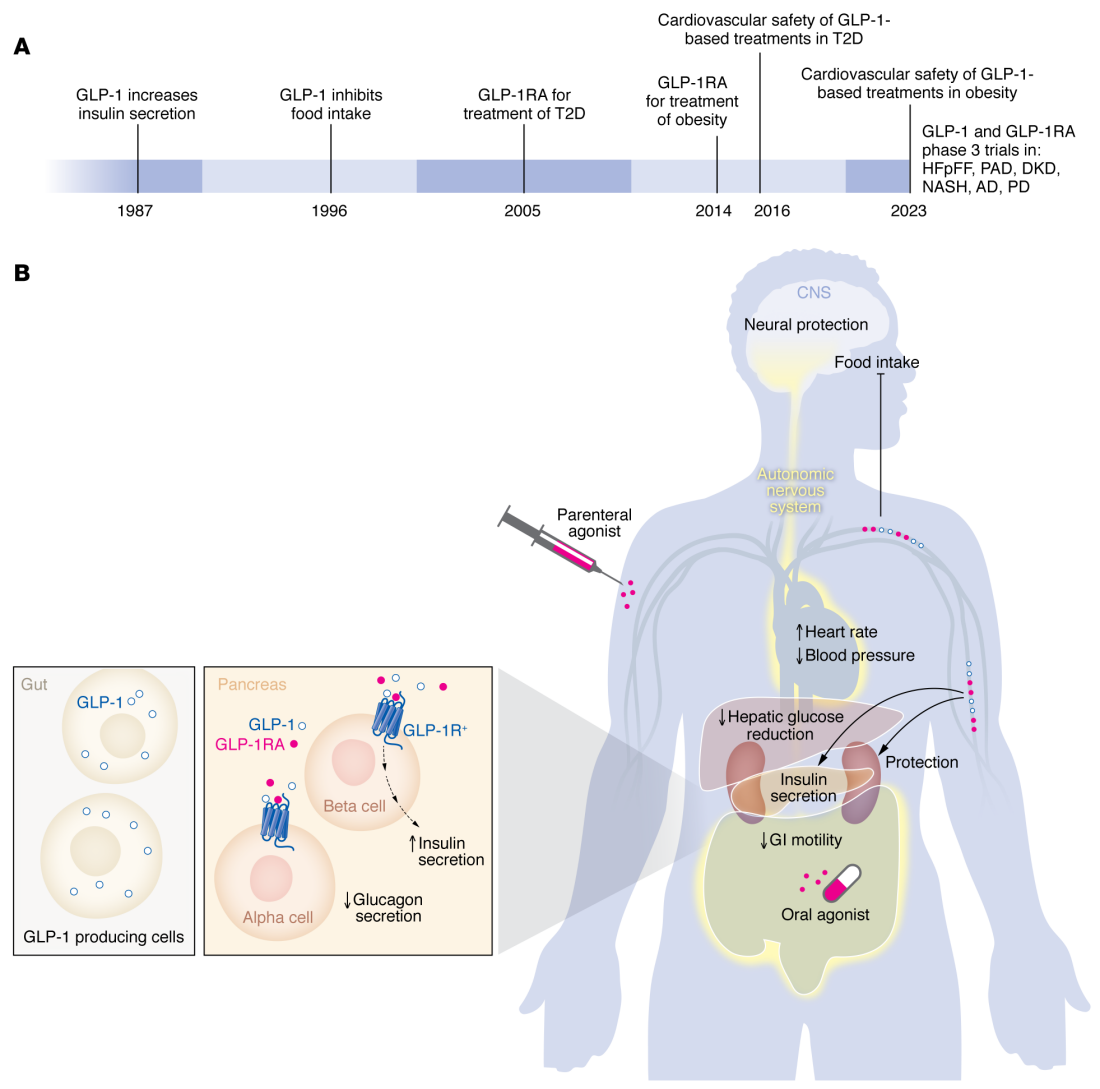
Translational GLP-1 science continues to improve options for individuals with chronic cardiometabolic disorders. (**A**) Data revealing GLP-1–stimulated insulin secretion supported the approval of the first GLP-1RA for the treatment of T2D in 2005. Nine years later, the link between GLP-1 and reduced food intake resulted the development and approval of the first GLP-1RA for obesity. The cardiovascular safety of GLP-1–based medicines was first demonstrated in T2D in 2016, and in people with obesity in 2023. GLP-1RA are currently being studied in phase 3 trials for the treatment of HFpEF, peripheral artery disease (PAD), diabetic kidney disease (DKD), metabolic liver disorders such as nonalcoholic steatohepatitis (NASH), and neurodegenerative disorders. (**B**) GLP-1 and GLP-1RAs act through the GLP-1R, which is a G protein coupled receptor. GLP-1 potentiates glucose-dependent insulin secretion in β-cells. The GLP-1R is also widely expressed in multiple tissues including several regions of the brain. Notably, activation of GLP-1R^^+^^ neurons in the hypothalamus and brainstem reduces food intake and promotes weight loss. GLP-1R^^+^^ neurons in the hindbrain also suppress hepatic glucose production. Further, GLP-1R influences BP and HR via the autonomic nervous system.

Clinically, GLP-1RAs are used to treat people with T2D and/or obesity (Figure 1), based on mechanisms described above linked to control of insulin and glucagon secretion, as well as reduction of food intake. Initial reports in animals showed that GLP-1 acutely increases blood pressure (BP) and heart rate (HR) in rats and mice, actions mediated through activation of the autonomic nervous system, including medullary catecholamine neurons, providing input to sympathetic preganglionic neurons (13). In humans, GLP-1R agonism frequently reduces BP; however, increases in HR are common and may be sustained with prolonged GLP-1R agonism. Notably, GLP-1RAs were subsequently shown to produce cardioprotective actions in animals (1). Importantly, starting in 2016, the first in a series of cardiovascular outcome studies demonstrated that long-acting GLP-1RAs reduce the rates of myocardial infarction, stroke, cardiovascular death, and all-cause mortality in people with T2D (1). More recent studies have extended the cardiovascular benefits of GLP-1R agonism to people with obesity, and subjects with heart failure and preserved ejection fraction (HFpEF).

Intriguingly, GLP-1RAs are neuroprotective in animals and several trials have examined the actions of exenatide in people with Parkinson’s disease (1). Aviles-Olmos and colleagues examined the effects of twice-daily exenatide over 12 months in a randomized controlled trial of people with Parkinson’s disease (PD) (14). Modest but detectable improvements were noted in PD activity scores and dementia rating scales; however, the small number of subjects studied (44 in total, 20 randomized to exenatide) and the limited duration of the trial limits definitive conclusions from being drawn.

## The future of GLP-1–based medicines

There are currently multiple once-weekly GLP-1RAs used to treat T2D, and two, liraglutide and semaglutide, are approved for therapy of people with obesity. A GIP-GLP-1R coagonist is now used to treat people with T2D and produces substantial weight loss, resulting in its approval for treatment of obesity in 2023. Oral semaglutide is also available as a once daily option, and several small molecule GLP-1R agonists, exemplified by orforglipron are in late stage clinical development (Figure 1). Newer GLP-1RAs and GLP-1–based coagonists also appear promising and are being studied in separate trials for T2D, diabetic kidney disease, peripheral artery disease, and metabolic liver disease (Figure 1). GLP-1RAs such as semaglutide have proven efficacy in HFpEF and are being studied in people with PD as well as in trials for Alzheimer’s disease. Hence, the expanding role of GLP-1-based medicines, together with newer more powerful GLP-1-based medicines (Figure 1), holds great promise for achieving improved health for substantial populations of individuals living with the complications of chronic cardiometabolic disorders.

## Version 1 01/16/2024

Electronic publication
